# High incidence of SV40-like sequences detection in tumour and peripheral blood cells of Japanese osteosarcoma patients

**DOI:** 10.1054/bjoc.2000.1213

**Published:** 2000-04-27

**Authors:** H Yamamoto, T Nakayama, H Murakami, T Hosaka, T Nakamata, T Tsuboyama, M Oka, T Nakamura, J Toguchida

**Affiliations:** Institute for Frontier Medical Sciences and Department of Orthopaedic Surgery, Faculty of Medicine, Kyoto University, Kyoto, 606-8507, Japan; Department of Orthopaedic Surgery, Kyoto City Hospital, Kyoto, 604-8845, Japan

**Keywords:** SV40, viral involvement, osteosarcoma

## Abstract

Recent studies have revealed the evidence for the significance of SV40 genome in human malignancies. In this paper, the presence of SV40-like sequences was investigated in 54 Japanese osteosarcomas in which mutations of the retinoblastoma (Rb), p53, MDM2, and CDK4 genes had been already analysed. Using polymerase chain reaction and Southern hybridization, SV40-like sequences were detected in 25 cases (46.3%). In most cases, only a part of SV40 genome was detected, and the regulatory region containing enhancer sequences was most frequently found (21/54, 38.9%). There was no apparent relationship between the presence of SV40-like sequences and tumour suppressor genes mutations in each tumour. The SV40-like sequences were also detected in peripheral blood cells of substantial proportion of the patients (43.3%), whereas the incidence was much lower (4.7%) in normal healthy controls. This difference is statistically highly significant (*P*< 0.0001), suggesting that the presence of SV40-like sequences, even if only a part, may play some roles to predispose individuals to osteosarcoma. © 2000 Cancer Research Campaign


British Journal of Cancer (2000) **82**(10), 1677–1681
© 2000 Cancer Research Campaign
DOI: 10.1054/ bjoc.2000.1213, available online at http://www.idealibrary.com on IDEAL®

### High incidence of SV40-like sequences detection in tumour and peripheral blood cells of Japanese osteosarcoma patients

**H Yamamoto[1,2], T Nakayama[3], H Murakami[1,2], T Hosaka[1,2], T Nakamata[1,2], T Tsuboyama[2], M Oka[1], T Nakamura[2] and J Toguchida[1,2]**

[1]Institute for Frontier Medical Sciences, and [2]Department of Orthopaedic Surgery, Faculty of Medicine, Kyoto University, Kyoto, 606-8507, Japan;
[3]Department of Orthopaedic Surgery, Kyoto City Hospital, Kyoto 604-8845, Japan

**Summary** Recent studies have revealed the evidence for the significance of SV40 genome in human malignancies. In this paper, the presence of SV40-like sequences was investigated in 54 Japanese osteosarcomas in which mutations of the retinoblastoma (Rb), p53, MDM2, and CDK4 genes had been already analysed. Using polymerase chain reaction and Southern hybridization, SV40-like sequences were detected in 25 cases (46.3%). In most cases, only a part of SV40 genome was detected, and the regulatory region containing enhancer sequences was most frequently found (21/54, 38.9%). There was no apparent relationship between the presence of SV40-like sequences and tumour suppressor genes mutations in each tumour. The SV40-like sequences were also detected in peripheral blood cells of substantial proportion of the patients (43.3%), whereas the incidence was much lower (4.7%) in normal healthy controls. This difference is statistically highly significant ($P < 0.0001$), suggesting that the presence of SV40-like sequences, even if only a part, may play some roles to predispose individuals to osteosarcoma. © 2000 Cancer Research Campaign

**Keywords:** SV40; viral involvement; osteosarcoma

Osteosarcoma is the most frequent malignant bone tumour second to multiple myeloma. Molecular analyses have revealed the presence of somatic mutations in two tumour suppressor genes, the retinoblastoma (Rb) and p53 genes, in more than 60% of cases respectively (Toguchida et al, 1992; Wadayama et al, 1994). In addition, hereditary mutations of these genes predispose individuals to osteosarcomas, suggesting the important roles of these mutations in the development of this type of tumour (Friend et al, 1987; Malkin et al, 1990). Mutations of genes which are functionally related to the Rb or p53 genes were found in some tumours with no apparent mutations of the Rb or p53 genes themselves, indicating the presence of alternative mechanisms to inactivate the tumour suppressor functions (Oliner et al, 1992; Khativ et al, 1993). There are, however, some tumours without any detectable mutations in molecules on either the Rb- or p53-pathway. These tumours may have some other genetic alterations which are equivalent with loss-of-function mutations of the tumour suppressor genes.

Simian virus 40 (SV40) is a member of poliomavirus, which is semipermissive in the human cells; the virus could both replicate in and transform human cells (Fanning and Knippers, 1992). The ability of SV40 to transform cells depends mainly on the function of large T antigen (Tag), which makes complexes with several tumour suppressor proteins, such as Rb and p53 and induce cell division (Fanning and Knippers, 1992). The first evidence for the presence of SV40-like sequences in human tumours was shown in brain tumours (Bergsagel et al, 1992). The results were confirmed by reports from other laboratories (Lednicky et al, 1995; Martini et al, 1996), and SV40-like sequences were also found in mesotheliomas (Carbone et al, 1994), bone tumours (Carbone et al, 1996) and papillary thyroid carcinomas (Pacini et al, 1998), all of which were tumours found in SV40-infected or transgenic rodents (Diamandopoulos, 1972; Ledent et al, 1991; Cicala et al, 1993). In the case of osteosarcomas, however, the incidence of SV40-like sequences varied greatly depending on the sample sources (Carbone et al, 1996). In the case of mesothelioma, it was shown that the presence of SV40-like sequence resulted in the expression of Tag protein, which binds to p53 (Carbone et al, 1997). No data, however, were reported whether the Tag was expressed in osteosarcoma tumours containing SV40-like sequences. There is also a discordance about the incidence of SV40-like sequences in normal tissues. In the initial report by Bergsagel et al no SV40-like sequences was detected in 100 blood specimens from normal controls (Bergsagel et al, 1992), whereas Martini et al found SV40-like sequences in 23% of peripheral blood samples from normal individuals (Martini et al, 1996). No data were presented concerning this point in osteosarcoma patients, and it will be intriguing to compare the incidence of SV40-like sequences in peripheral blood cells between patients and healthy controls, which may address the role of SV40-like sequences in osteosarcomas.

In this report, we have addressed the above mentioned issues using Japanese osteosarcoma samples, in which we have already performed intensive DNA-based analyses for tumour suppressor gene mutations.

*Received 12 October 1999*
*Revised 6 January 2000*
*Accepted 17 January 2000*

*Correspondence to:* J Toguchida, Institute for Frontier Medical Sciences, Kyoto University, Kyoto 606-8507, Japan

### MATERIALS AND METHODS

### DNA extraction

Osteosarcoma tumour tissues were obtained at the time of surgery from 54 patients whose age ranged from 3 to 25 years at the diagnosis (mean age 15.8 years). All patients received the preoperative chemotherapy by the combination of two or three anticancer drugs including doxorubicin, cis-platinum and methotrexate. Tumour tissues were quickly frozen and kept in –80°C until DNA extraction. Whole blood sample was collected from the corresponding patients in 30 cases before or after the surgery of the primary lesion, and also from 64 healthy volunteers (mean age 24.4 years) who were born after 1965. High-molecular-weight-DNAs were extracted from tumour tissues and whole blood cells as previously described (Sambrook et al, 1989).

### PCR and Southern blot analysis

DNAs were extracted from each sample, and 100 ng of DNAs were used as templates for polymerase chain reaction (PCR) containing 10 pmol of each primer, 0.2 mM dNTP, 1.5–2.5 mM of magnesium chloride and 0.1 units of *rTaq* polymerase (Toyobo, Shiga, Japan). After completion of the first 30 cycles, 0.1 units of *rTaq* was added, and another 30 cycles of amplifications were performed. Primers to amplify each region of SV40 used were: for a 315 bp region of the regulatory region (R region), R1 (AATGTGTGTCAGTTAGGGTGTG) and R2 (TCCAAAAAAGCCTCCTCACTACTT); for a 574 bp region encompassing the Rb-pocket binding domain of Tag (N region), SVfor2 (CTTTGAGGCTTCTGGGATGCAACT), and SVrev (GCATGACTCAAAAAACTTAGCAATTCTG); for a 443 bp region of the carboxyl-terminal domain of Tag (C region), T1 (GACCTGTGGCTGAGTTTGCTCA) and T2 (GCTTTATTTGTAACCATTATAAG). Sequences of these primers were derived from a previously published study (Lednicky et al, 1995). The PCR products were electrophoresed in 2% agarose gel, transferred to nylon membrane (Hybond-N+, Amersham Pharmacia Biotech), and hybridized with $^{32}P$-labelled fragments generated by PCR using same primers from DNA of 509SV, which is a SV40-transfected human liposarcoma cell line (T Nakayama, unpublished data). The sequences of these fragments were confirmed by sequencing and were found to be identical with those of the published SV40 genome (Reddy et al, 1978) (data not shown). We repeated these experiments using internal oligonucleotide primers used in a previous study (Carbone et al, 1996), and obtained the same results (data not shown).

**Table 1** SV40-like sequences in osteosarcoma samples

| Regions positive for SV40-like sequences[a] | No. of cases |
|---|---|
| R+N+C | 1 |
| R+N | 2 |
| R+C | 2 |
| R | 16 |
| N+C | 1 |
| N | 1 |
| C | 2 |
| Total positive/total samples | 25/54 (47%) |

[a]R, regulatory region; N, amino-terminal region containing the Rb-pocket

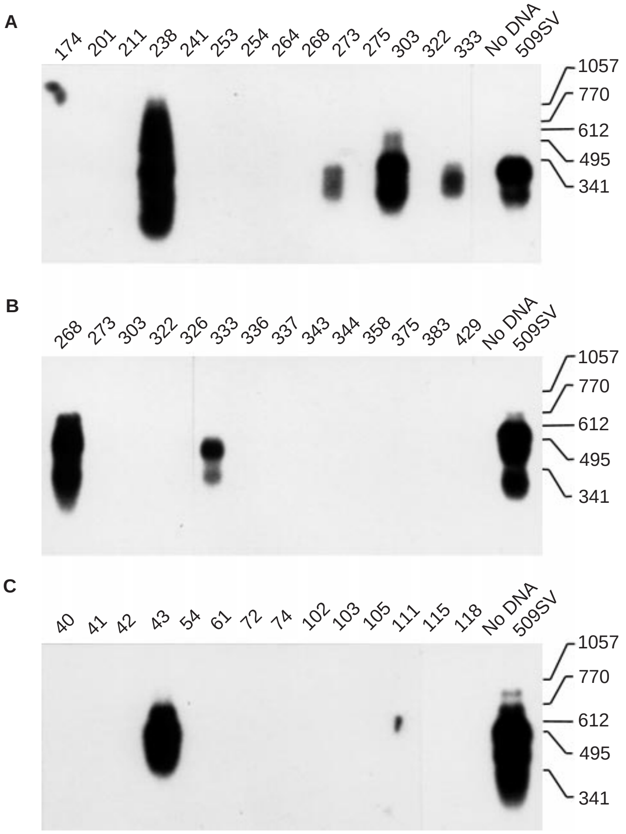

**Figure 1** Detection of SV40-like sequences in osteosarcoma tumours. Numbers on the top of each lane indicate the ID number of each sample. No DNA indicates PCR products without template DNA, and 509SV indicates PCR products using DNA isolated from a liposarcoma cell line transfected with the expression vector containing the replication defective SV40. (**A**) the regulatory (R) region; (**B**) the amino-terminal (N) region containing the Rb-pocket domain; (**C**) the carboxyl-terminal (C) region. Primers for each PCR and probes for hybridization are described in Materials and Methods section. Numbers on the right side indicate the position of each fragment of *Hinc*II digested φX174 DNAs

### RESULTS

### Presence of SV40-like sequences in osteosarcoma tumours

In 54 tumour samples, 25 tumours (46.3%) were found to contain at least one of the three regions of SV40-like sequences (Figure 1 and Table 1). In most cases, the size of amplified SV40-like sequences was identical with that of control fragments which were amplified using DNA from a SV40-transfected cell line, but some samples showed fragments with a slightly different size (for example, sample 238 in Figure 1A). Most of the fragments were detected only after Southern blot analyses, but some were visible in the ethidium-bromide-staining gel after the second round of PCR. One fragment amplified by using primers specific to the regulatory (R) region and another fragment amplified by using primers specific to the amino-terminal (N) region including the ‘Rb-pocket’ binding domain were gel-purified and directly sequenced. The sequences of both fragments were completely identical with the corresponding authentic sequences of SV40 genome (Reddy et al, 1978 and data not shown).

 *© 2000 Cancer Research Campaign*

**Table 2** Relationship between the status of tumour suppressor genes and the presence of SV-40 like sequences

| Genes | Mutation status | SV-40 like sequences | | |
|---|---|---|---|---|
| | | No. of positive cases | No. of negative cases | % of positive cases |
| Rb | wt | 5 | 8 | 38.5 |
| | mut | 9 | 10 | 47.4 |
| p53 | wt | 7 | 6 | 53.8 |
| | mut | 7 | 12 | 36.8 |
| Rb/p53 | wt/wt | 3 | 2 | 60.0 |
| | wt/mut | 2 | 6 | 25.0 |
| | mut/wt | 4 | 4 | 50.0 |
| | mut/mut | 5 | 6 | 45.5 |

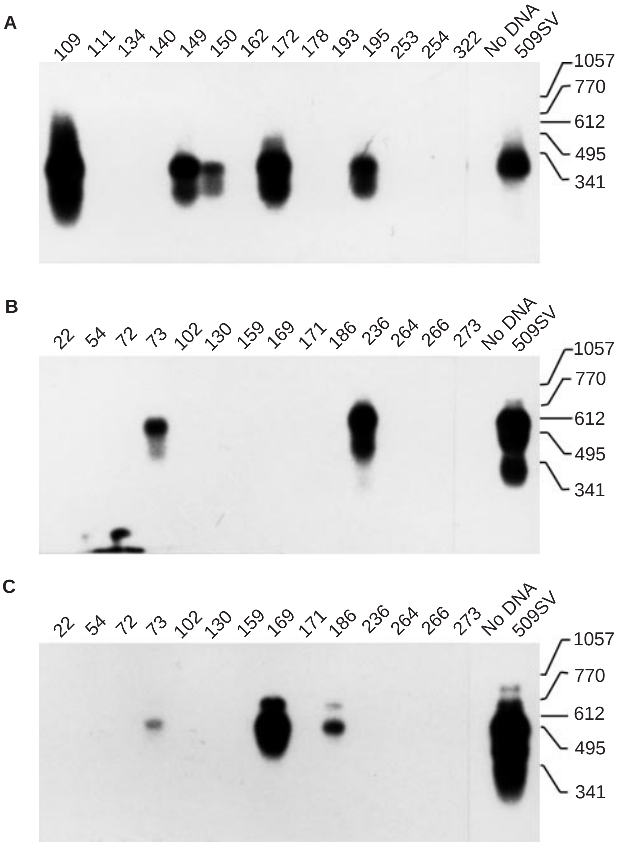

**Figure 2** Detection of SV40-like sequences in peripheral blood cells from osteosarcoma patients. See details in the legend for Figure 1. (**A**) The regulatory (R) region; (**B**) the amino-terminal (N) region containing the Rb-pocket domain; (**C**) the carboxyl-terminal (C) region

Among 25 samples with SV40-like sequences, only one case showed amplified fragments from all of the three regions, while amplified fragments were detected only in one or two of three regions in other 24 samples (Table 1). Fragments from the R region were found most frequently (21/54, 38.9%), followed by carboxyl-terminal (C) region (6/54, 11.1%), and the frequency of the N region was the lowest (5/54, 9.3%). There were two cases in which only R and C regions, but not N region were amplified (Table 1).

### Relationship with the status of genes on the Rb- and p53 gene pathways

Mutations analyses of the Rb, p53, MDM2, and CDK4 genes had been performed in 32 of 54 osteosarcomas used in this study, and the results were reported previously (Toguchida et al, 1992; Wadayama et al, 1994; Nakayama et al, 1995; Kanoe et al, 1998). The status of the Rb gene was defined as Rb(mut) for a tumour with a mutation in the Rb gene or amplification of the CDK4 gene (19 cases), and other tumours were defined as Rb(wt) (13 cases) (Table 2). In the case of the p53 gene, tumours were defined as p53(mut) for a tumour with a mutation in the p53 gene or amplification of the MDM2 gene (19 cases), and others were defined as p53(wt) (13 cases). The incidence of SV40-like sequences in tumours with Rb(wt) and Rb(mut) showed no significant difference (38.5% vs 47.4%; $P = 0.618$, $\chi^2$ test). The status of the p53 gene also showed no significant relationship with the incidence (53.8% vs 36.8%; $P = 0.341$, $\chi^2$ test). Furthermore, 32 cases were categorized into four groups based on the status of Rb and p53 genes; Rb(wt)/p53(wt) in five cases, Rb(wt)/p53(mut) in eight cases, Rb(mut)/p53(wt) in eight cases, and Rb(mut)/p53(mut) in 11 cases (Table 2). Similar to the results of the analyses for each gene, there was no statistically significant difference in the incidence of SV40-like sequences among these groups ($P = 0.611$, $\chi^2$ test).

### Presence of SV40-like sequences in peripheral blood cells DNA

DNAs from peripheral blood cells (PBCs) were available from 30 patients among 54 cases in this study, and SV40-like sequences were detected in 13 cases (43.3%) (Figure 2 and Table 3). The incidence of R region was the highest (7/30, 23.3%) as same as the results in tumours, followed by the C region (6/30, 20%) and the N region (3/30, 10%). The presence of SV40-like sequences in tumour samples was not apparently associated with that in the corresponding PBCs (Table 4). SV40-like sequences were amplified from PBCs but not from tumours in five patients, and only two cases among eight in which SV40-like sequences were detected in both tumour and PBC DNAs showed an identical pattern of amplified regions. Among the 64 age-matched healthy volunteers, only three (4.7%) were found to have SV40-like sequences in PBC genome (Table 3), and the difference in incidence between the patient and control group was statistically highly significant ($P < 0.0001$, $\chi^2$ test).

*© 2000 Cancer Research Campaign* 

**Table 3** SV40-like sequences in peripheral blood cells

| Regions positive for SV40-like sequences[a] | No. of cases | |
|---|---|---|
| | Osteosarcoma patients | Healthy controls |
| R+N+C | 0 | 0 |
| R+N | 1 | 0 |
| R+C | 1 | 0 |
| R | 5 | 0 |
| N+C | 1 | 0 |
| N | 1 | 1 |
| C | 4 | 2 |
| Total positive/total samples | 13/30 (43.3%) | 3/64 (4.7%) |

[a]R, regulatory region; N, amino-terminal region containing the Rb-pocket binding domain; C, carboxyl-terminal region.

**Table 4** SV-40 like sequences in tumours and peripheral blood cells of osteosarcoma patients

| SV40-like sequences in tumours | SV40-like sequences in PBCs | |
|---|---|---|
| | No. of positive cases | No. of negative cases |
| Positive | 8 | 8 |
| Negative | 5 | 9 |

### DISCUSSION

The incidence of SV40-like sequences in osteosarcoma tumours in this study (25/54, 46.3%) is compatible with previous three reports: 40/126 (31.7%), 5/10 (50%), and 9/35 (25.7%) (Carbone et al, 1996; Lednicky et al, 1997; Mendoza et al, 1998). In the case of mesothelioma, the expression of Tag protein was detected by Western blot analyses and immunohistochemical studies, indicating the presence of the entire coding region of Tag in tumour cells (Carbone et al, 1997). In the case of osteosarcomas in this study, however, only a part of Tag genome was detected in most cases, and most of the fragments were detected only by Southern blot analyses after 60 cycles of amplification, suggesting that the SV40-like sequences were not present in each tumour cell. We have no clear explanation for our detection of only a part of SV40 genome in most cases. Severe tumour necrosis due to the preoperative chemotherapy may cause the degradation of DNA, which may affect the sensitivity of detection. Most of DNA samples, however, were able to be used in Southern blot analyses of the Rb or p53 genes, which require high-molecular-weight DNA with much better quality than those required in the PCR analyses. Therefore, the reason for the failure to detect the entire region of SV40 genome was not merely due to the poor quality of DNA. Since genomic instability is one of the important characteristics of malignant cells, it is not unlikely that the SV40 genome might be rearranged after exerting its role in earlier stages of multistep tumorigenesis in osteosarcomas, and the observed results are merely reminiscent of such events, as proposed in the case of papilloma virus infection in cervical cancers (zur Hausen, 1996). Alternatively, the presence of a part of SV40-like sequences itself may have some mechanistic role to play. The most frequently detected region in SV40 genome in this study is the regulatory region sequence (21/54 in tumour samples). The sequence of this region contains a transcription enhancer element which is able to activate the expression of genes in either orientation (Fanning and Knippers, 1992), and it was shown that the integrated sequence of this region produced fusion transcripts in SV40-infected human keratinocytes (Chen et al, 1996). Therefore, some genes flanking the integrated SV40 sequences are possibly up-regulated to endow cells with enhanced proliferation activity, although we did not confirm whether SV40 genome detected in our samples was integrated into the genomic DNA.

There is no definite correlation between the presence of SV40-like sequences and mutations of tumour suppressor genes in our samples. Mendoza et al also found no correlation of the presence of Tag-like sequences with tumour suppressor inactivation in osteosarcomas (Mendoza et al, 1998). These results are in contrast with those of mesotheliomas, in which no p53 gene mutation was detected in most cases with the expression of Tag (Carbone et al, 1997). SV40-like sequences in osteosarcomas might not be related with the function of Tag as an inactivator of tumour suppressor genes.

The major source of human exposure to SV40 is considered to be the contaminated vaccines for poliomyelitis (Shah and Nathanson, 1976). In 1961, shortly after the discovery of SV40 (Sweet and Hilleman, 1960), mass administration of Sabin vaccine was carried out in Japan, because of an outbreak of poliomyelitis in the previous year (5606 cases in 1960) (Takatsu et al, 1973). In the following 2 years, 1962 and 1963, mass vaccination was also performed and almost all Japanese children from 3 months to 12 years of age received live oral vaccine imported from Canada and USSR, which might have had a higher titre of SV40 contamination (Shah and Nathanson, 1976). Therefore, although the length of period when the contaminated vaccine was used was much shorter than in the USA, a considerable fraction of people in Japan at that age were at risk of infection by SV40. None of the individuals in this study, however, either patients or healthy controls, were born before 1965, and therefore they should have had the lowest risk of receiving contaminated vaccine. Nevertheless, the SV40-like sequences were present in tumour and also in normal cells, suggesting the unknown mechanisms for SV40 to spread over the population.

In terms of the presence of SV40-like sequences in PBCs, our results agreed with the data by Martini et al using brain tumour cases (Martini et al, 1996), and disagreed with the data by Bergsagel et al (1992), although we have no clear explanation of this discrepancy. The difference of incidence of SV40-like sequences between osteosarcoma patients and healthy volunteers is quite an intriguing matter. Because PBCs were collected from patients in tumour-bearing condition in most cases, there is a possibility that the origin of amplified SV40-like sequences in patients is not from peripheral leucocytes, but from circulating tumour cells. However, SV40-like sequence was also detected from PBCs collected from patients in an apparently tumour-free condition after tumour resection. There was poor correlation between finding SV40-like sequence in tumour tissue and in PBCs in each patient. Although viral involvement may occur at the same time in all tissues, the process to maintain or eliminate the viral genome may be different in each type of tissue, causing different patterns of the reminiscence of SV40 sequences. Although there were such perplexing data to be resolved, the difference of incidence of SV40-like sequences was clear between PBCs from osteosarcoma patients and control individuals, suggesting some role for

*© 2000 Cancer Research Campaign*

SV40-like sequences in the development of osteosarcoma. Further nationwide studies may provide us with the answer to this question.

### ACKNOWLEDGEMENTS

We appreciate the technical assistance of Mika Tanaka and Junko Tsujimoto, and blood sample volunteers for their kind cooperation.

### REFERENCES

Bergsagel DJ, Finegold MJ, Butel JS, Kupsky WJ and Garcea RL (1992) DNA sequences similar to those of simian virus 40 in ependymomas and choroid plexus tumors of childhood. *N Engl J Med* **326**: 988–993

Carbone M, Pass HI, Rizzo P, Marinetti MR, Di Muzio M, Mew DJY, Levine AS and Procopio A (1994) Simian virus 40-like sequences in human pleural mesothelioma. *Oncogene* **9**: 1781–1790

Carbone M, Rizzo P, Procopio A, Giuliano MT, Pass HI, Gebhardt MC, Mangham C, Hansen M, Malkin DF, Bushart G, Pompetti F, Picci P, Levine AS, Bergsagel JD and Garcea RL (1996) SV40-like sequences in human bone tumors. *Oncogene* **13**: 527–535

Carbone M, Rizzo P, Grimley PM, Procopio A, Mew DJY, Shridhar V, de Bartolomeis A, Esposito V, Giuliano MT, Steinberg SM, Levine AS, Giordano A and Pass HI (1997) Simian virus-40 large-T antigen binds p53 in human mesotheliomas. *Nat Med* **3**: 908–912

Chen G, Baez JM and Steinberg ML (1996) Integration of the SV40 promoter/enhancer sequences in an anchorage-independent clonal subline of SV40-infected human keratinocytes. *Gene* **183**: 41–45

Cicala C, Pompetti F and Carbone M (1993) SV40 induces mesotheliomas in hamsters. *Am J Pathol* **142**: 1524–1533

Diamandopoulos GT (1972) Leukemia, lymphoma, and osteosarcoma induced in the Syrian golden hamster by simian virus 40. *Science* **176**: 173–175

Fanning E and Knippers R (1992) Structure and function of simian virus 40 large tumor antigen. *Annu Rev Biochem* **61**: 55–85

Friend SH, Bernards R, Rogelj R, Weinberg RA, Rapaport JM, Albert DM and Dryja TP (1987) A human DNA segment with properties of the gene that predisposes to retinoblastoma and osteosarcoma. *Nature* **323**: 643–646

Kanoe H, Nakayama T, Murakami H, Hosaka T, Yamamoto H, Nakashima Y, Tsuboyama T, Nakamura T, Sasaki MS and Toguchida J (1998) Amplification of the CDK4 gene in sarcomas: tumor specificity and relationship with the RB gene mutation. *Anticancer Res* **18**: 2317–2322

Khatib ZA, Matsushime H, Valentine M, Shapiro DN, Sherr CJ and Look AT (1993) Coamplification of CDK4 gene with MDM2 and GLI in human sarcomas. *Cancer Res* **53**: 5535–5541

Ledent C, Dumont J, Vassart G and Parmentier M (1991) Thyroid adenocarcinomas secondary to tissue-specific expression of simian virus-40 large T-antigen in transgenic mice. *Endocrinology* **129**: 1391–1401

Lednicky JA, Garcea RL, Bergsagel DJ and Butel JS (1995) Natural simian virus 40 strains are present in human choroid plexus and ependymoma tumors. *Virology* **212**: 710–717

Lednicky JA, Stewart AR, Jenkins JJ III, Finegold MJ and Butel JS (1997) SV40 DNA in human osteosarcomas shows sequence variation among T-antigen genes. *Int J Cancer* **72**: 791–800

Malkin D, Li FP, Strong LC, Fraumeni Jr JF, Nelson CE, Kim DH, Kassel J, Gryka MA, Bichoff FZ, Tainsky MA and Friend SH (1990) Germ line p53 mutations in a familial syndrome of breast cancer, sarcomas, and other neoplasms. *Science* **250**: 1233–1238

Martini F, Iaccheri L, Lazzarin L, Carinci P, Corallini A, Gerosa M, Iuzzolino P, Barbanti-Brodano G and Tognon M (1996) SV40 early region and large T antigen in human brain tumors, peripheral blood cells, and sperm fluids from healthy individuals. *Cancer Res* **56**: 4820–4825

Mendoza SM, Konishi T and Miller CW (1998) Integration of SV40 in human osteosarcoma DNA. *Oncogene* **17**: 2457–2462

Nakayama T, Toguchida J, Wadayama B, Kanoe H, Kotoura Y and Sasaki MS (1995) MDM2 gene amplification in bone and soft-tissue tumors: Association with tumor progression in differentiated adipose-tissue tumors. *Int J Cancer* **64**: 342–346

Oliner JD, Kinzler KW, Meltzer PS, George DL and Vogelstein B (1992) Amplification of a gene encoding a p53-associated protein in human sarcomas. *Nature* **358**: 80–83

Pacini F, Vivaldi A, Santoro M, Fedele M, Fusco A, Romei C, Basolo F and Pinchera A (1998) Simian virus 40-like DNA sequences in human papillary thyroid carcinomas. *Oncogene* **16**: 665–669

Reddy VB, Thimmappaya B, Dhar R, Subramanian KN, Zain BS, Pan J, Ghosh PK, Celma ML and Weissman SM (1978) The genome of simian virus 40. *Science* **200**: 494–502

Sambrook J, Fritsch EF, Maniatis T (eds) (1989) *Molecular cloning: a laboratory manual* (2nd ed.), Cold Spring Harbor Press, Cold Spring Harbor, NY

Shah K and Nathanson N (1976) Human exposure to SV40: review and comment. *Am J Epidemiol* **103**: 1–12

Sweet BH and Hilleman MR (1960) The vacuolating virus, S.V.$_{40}$. *Proc Soc Exp Biol Med* **105**: 420–427

Takatsu T, Tagaya I and Hirayama M (1973) Poliomyelitis in Japan during the period 1962–68 after the introduction of mass vaccination with Sabin vaccine. *Bull Wld Hlth Org* **49**: 129–137

Toguchida J, Yamaguchi T, Ritchie B, Beauchamp RL, Dayton SH, Herrera GE, Yamamuro T, Kotoura Y, Sasaki MS, Little JB, Weichselbaum RR, Ishizaki K and Yandell DW (1992) Mutation spectrum of the p53 gene in bone and soft tissue sarcomas. *Cancer Res* **52**: 6194–6199

Wadayama B, Toguchida J, Shimizu T, Ishizaki K, Sasaki MS, Kotoura Y and Yamamuro T (1994) Mutation spectrum, of the retinoblastoma gene in osteosarcomas. *Cancer Res* **54**: 3042–3048

zur Hausen H (1996) Papillomavirus infections – a major cause of human cancers. *Biochem Biophys Acta* **1288**: F55–F78

© 2000 Cancer Research Campaign
